# Push by a net, pull by a cow: can zooprophylaxis enhance the impact of insecticide treated bed nets on malaria control?

**DOI:** 10.1186/1756-3305-7-52

**Published:** 2014-01-28

**Authors:** Hanako Iwashita, Gabriel O Dida, George O Sonye, Toshihiko Sunahara, Kyoko Futami, Sammy M Njenga, Luis F Chaves, Noboru Minakawa

**Affiliations:** 1 Department of Vector Ecology and Environment, Institute of Tropical Medicine (NEKKEN), Nagasaki University, Nagasaki, Japan; 2 ASK Community Project, Mbita, Kenya; 3 Eastern and Southern Africa Centre of International Parasite Control, Nairobi, Kenya; 4 Programa de Investigación en Enfermedades Tropicales (PIET), Escuela de Medicina Veterinaria, Universidad Nacional, Heredia, Costa Rica

**Keywords:** *Anopheles*, Bed net, Bloodfeeding, *Plasmodium falciparum*, Zooprophylaxis

## Abstract

**Background:**

Mass insecticide treated bed net (ITN) deployment, and its associated coverage of populations at risk, had “pushed” a decline in malaria transmission. However, it is unknown whether malaria control is being enhanced by zooprophylaxis, i.e., mosquitoes diverted to feed on hosts different from humans, a phenomenon that could further reduce malaria entomological transmission risk in areas where livestock herding is common.

**Methods:**

Between May and July 2009, we collected mosquitoes in 104 houses from three neighboring villages with high ITN coverage (over 80%), along Lake Victoria. We also performed a census of livestock in the area and georeferenced tethering points for all herds, as well as, mosquito larval habitats. Bloodmeal contents from sampled mosquitoes were analyzed, and each mosquito was individually tested for malaria sporozoite infections. We then evaluated the association of human density, ITN use, livestock abundance and larval habitats with mosquito abundance, bloodfeeding on humans and malaria sporozoite rate using generalized linear mixed effects models.

**Results:**

We collected a total of 8123 mosquitoes, of which 1664 were *Anopheles* spp. malaria vectors over 295 household spray catches. We found that vector household abundance was mainly driven by the number of householders (P < 0.05), goats/sheep tethered around the house (P < 0.05) and ITNs, which halved mosquito abundance (P < 0.05). In general, similar patterns were observed for *Anopheles arabiensis*, but not *An. gambiae s.s.* and *An. funestus s.s.,* whose density did not increase with the presence of livestock animals. Feeding on humans significantly increased in all species with the number of householders (P < 0.05), and only significantly decreased for *An. arabiensis* in the presence of cattle (P < 0.05). Only 26 *Anopheles* spp. vectors had malaria sporozoites with the sporozoite rate significantly decreasing as the proportion of cattle feeding mosquitoes increased (P < 0.05).

**Conclusion:**

Our data suggest that cattle, in settings with large ITN coverage, have the potential to drive an unexpected “push-pull” malaria control system, where *An. arabiensis* mosquitoes “pushed” out of human contact by ITNs are likely being further “pulled” by cattle.

## Background

The dominant *Anopheles* spp. (Diptera: Culicidae) malaria vectors in East Africa are *An. gambiae s.s.* Giles and *An. funestus s.s.* Giles*,* which are regarded as highly anthropophagic (and endophagic), and *An. arabiensis* Patton, which is regarded as zoophagic (and exophagic) [1]. Insecticide treated nets (ITNs) have significantly improved malaria control in endemic areas such as Sub-Saharan Africa (SSA) [2], but also elsewhere, e.g., Vanuatu in Oceania [3,4]. ITNs alone have significantly reduced morbidity and mortality due to malaria in SSA [5], a fact that is probably connected with the observed decline of vector populations [6], especially *An. gambiae s.s.*[7,8]. Moreover, the larger the coverage, i.e., the more individuals sleeping under ITNs, the more effective ITNs seem to be, mainly because of the emergence of community effects [9,10].

However, the widespread emergence of insecticide resistance in African malaria vectors may hamper malaria control programs using ITNs [11,12]. Shifting biting hours and locations by *An. gambiae s.s.* after an increase of ITN coverage has become a strong concern [13,14]. This species is also known to become opportunistic by feeding on abundant hosts over “innately” preferred ones after an increase of ITNs [15]. Since ITNs are partially inefficient in the control of exophagic vectors like *An. arabiensis* and early-feeding vectors like *An. rivulorum* Leeson [16,17], these new phenomena call for integration of ITNs with a more robust, multi-faceted malaria control strategy [18].

ITNs are the major malaria control tool in Lake Victoria basin (LVB), western Kenya [19]. Nevertheless, despite a high ITN coverage in LVB [20], malaria prevalence remains hyperendemic (over 40% prevalence) [21]. This high prevalence could have been influenced by mosquito-mediated transmission happening outside households protected by ITNs [22-24]. The non-domiciliary transmission may ultimately reflect patterns of entomological risk for malaria transmission that could be further driven by the heterogeneity of house *Anopheles* spp. abundance [25-28].

An important factor likely underpinning adult mosquito abundance heterogeneity is their blood-feeding behavior [29]. For example, *An. arabiensis*, a species whose bloodmeals are not strongly biased towards humans [17], is currently the most abundant vector species within the *An. gambiae* complex in LVB, a trend that followed ITN use in western Kenya [7]. Within the limited distance that mosquitoes travel for bloodfeeding [30,31], mosquitoes may be attracted by any available vertebrate host [32], especially if their feeding is not biased [33]. Livestock breeding is a major economic activity in rural East Africa [34], providing abundant hosts that can serve as an alternative bloodmeal source for malaria vectors, especially as humans become unavailable blood sources because of ITN use [17,35]. More than a century ago, this phenomenon was called “zooprophylaxis” [36,37] a malaria control strategy where transmission is interrupted by attracting mosquitoes to dead-end hosts [38,39], and which has been long recommended by WHO as a protective measure against malaria [40].

**Figure 1:**
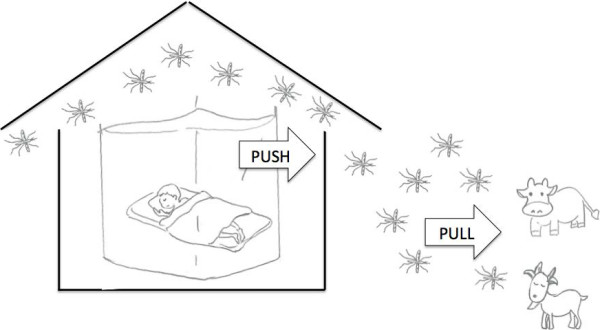
**Insecticide Treated Net (ITN) – Zooprophylaxis as a “Push – Pull” integrated malaria control strategy.** Mosquitoes entering the houses will either be killed or “pushed” out by ITNs, then they may be pulled further away by the presence of alternative hosts such as livestock.

Here we explored the potential of zooprophylaxis as an additional tool that could improve malaria control in endemic areas of East Africa under large ITN coverage. We hypothesized that zooprophylaxis can enhance the impacts of ITNs on malaria control by acting in a similar way to the “pull” component of a “push-pull” integrated pest management system [41]. Briefly, we consider that ITNs have worked as the “push” in malaria control, as supported by historical trends of decreased household mosquito abundance following the expansion of ITN coverage [6-8]. Then alternative hosts, livestock, kept at a certain distance from the households could serve as a “pull” to both keep mosquitoes away from humans and to waste their infective bites in dead end-hosts (Figure 1). We tested our hypothesis in three villages along the Kenyan shore of Lake Victoria, with a high ITN coverage.

## Methods

### Study area

**Figure 2:**
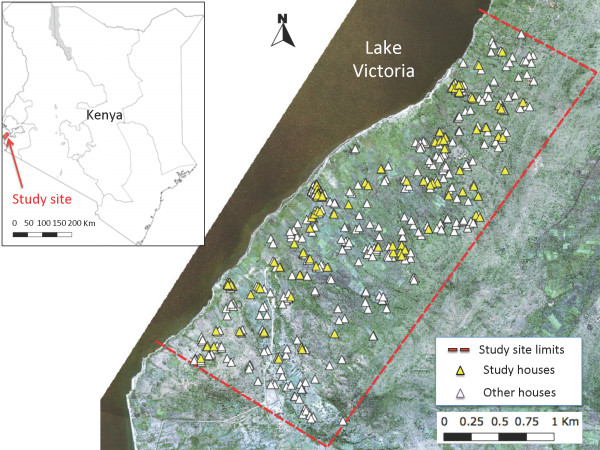
**Study Site.** This map shows the location of our study site in Kenya and the location of all the houses enrolled in our study and other neighboring houses enrolled in the Nagasaki University Health Demographic Survey System.

Our study site (Figure 2) comprised three villages, totaling 455 households over a 7.7 km^2^ surface in Lake Victoria shore, Mbita district, Nyanza province, Kenya (0°28’S and 34°11’E at the approximate center of the study area). Mbita’s rainfall pattern is bimodal, with a long (March - May) and a short (November - December) rainy seasons, all other months being relatively dry. Regardless of the season, the coastal lake environment maintains a high malaria vector abundance across seasons [42]. The high vector abundance in this area seems to drive year-round malaria transmission, with over 40% of the human population harboring *Plasmodium falciparum* (Welch) malaria parasites [21,43]. Most houses are built using a stick framework, which is plastered with a mixture of mud and cow dung and commonly covered with a corrugated iron roof or, in few instances, by thatched roofs [44]. Most residents belong to the Luo ethnic group, and depend on small-scale farming, fishing and livestock herding for subsistence. Cattle, goats and sheep are the main livestock species herd in the study area, while domestic animals are mainly chickens and dogs. The Luos sleep inside their houses with all domestic animals and livestock outside the house during the night-time, i.e., the time when *Anopheles* spp. malaria vectors commonly bloodfeed [29].

In 2006, Kenya’s National Malaria Control Programme began to introduce ITNs in our study area through government health facilities [45]. ITNs remain as the main malaria control tool in the area [46]. Currently, there is an estimated 80% ITN household coverage, where a household is considered to be ITN covered if there is at least one ITN for each two residents [44].

### Household survey

In May 2009, out of the 455 households in the study area, we chose 104 that were: (i) enrolled in the Health Demographic Surveillance System (HDSS) of Nagasaki University and Kenya Medical Research Institute and (ii) had iron or thatched roofs and eaves, and (iii) household heads of the houses were livestock breeders or took care of livestock belonging to people outside the study area (iv) where household heads provided informed consent for mosquito collection inside the houses. (ii) and (iii) were selection criteria included to minimize differences in socio-economic status among households, provided the relevant role that income differences have had in previous comprehensive studies on zooprophylaxis in Africa [47,48]. For the subsequent analysis, the floor area (m^2^) of each house was estimated using a metric tape. We used the floor area as a measurement of house size.

### Livestock census

We performed a census of all livestock in the study area, in order to estimate the availability of potentially zooprophilatic hosts. Specifically, we counted the number of cattle, goats and sheep, and georeferenced the night-time tethering location of each herd using a hand-held global positioning system (GPS; GPSmap 60CSx, Garmin International Inc., Olathe, USA). For large herds, those where animals were tethered in groups separated by more than 20 m, we separately recorded the location of each group. For the analysis we considered the combined number of goats and sheep, because they are herd and tether together. We also considered calf, juvenile goat and sheep to be equivalent to half adult, based on domestic animal traits (e.g., body surface area, body mass and CO_2_) that are relevant for mosquito bloodfeeding [49].

### Ethical clearance

This study was approved by the Ethical Committee of The Graduate School of International Health Development, Nagasaki University, and Kenya Medical Research Institute (KEMRI: SSC No. 1310). Informed consent was obtained from all heads of households after the study was explained in the local language.

### Mosquito larval habitat survey

Simultaneous to the household survey and livestock census, we examined occurrence of anopheline larvae in potential permanent and ephemeral habitats throughout the study area [42]. Two co-authors searched for anopheline larvae at each potential habitat for 10 minutes using standard mosquito dippers (350 ml; BioQuip Products, Rancho Dominguez, USA). A location of each positive habitat was recorded using a GPS. The locations of permanent habitats were obtained from a previous study [42]. We considered “permanent” habitats swamps or lagoons occurring along Lake Victoria shore and “ephemeral” as those generated by the impact of rainfall on the study area landscape topography. We also want to clarify that larval habitat separation into permanent and ephemeral could be relevant to understand differences in the potential recruitment of adult mosquito vectors in the area. While *An. arabiensis* and *An. gambiae s.s.* can thrive mainly in ephemeral habitat, *An funestus s.s.* is only successful at colonizing stable water bodies with aquatic vegetation [42].

### Adult mosquito sampling and ITN use patterns

Following the mosquito larval habitat survey, adult mosquito abundance and ITN use in the 104 study houses were surveyed once per month from May to July 2009. Indoor-resting mosquitoes were sampled using pyrethrum spray catches (PSC). For each PSC we laid down white sheets on the house floor and sprayed permethrin 0.5%, a synthetic pyrethroid insecticide (Doom^©^, Mortain Inc, Sharjah, UAE), inside the houses, from outside through the house eaves, a procedure done to minimize the possibility of mosquito escape via the eaves. After 10 minutes knocked down mosquitoes were collected from the white sheets and transported with ice to the Nagasaki University laboratory at ICIPE (International Centre of Insect Physiology and Ecology, Mbita, Kenya). The PSC was performed during the early morning hours (4:30 ~ 6:00 hours), by a team including the first author and 3 ~ 5 local assistants.

We estimated ITN use by counting the number of people who slept in each household and the number of people who slept under an ITN by direct observation during the mosquito survey. For subsequent analyses we considered people older than 15 years as adults, between 5 and 15 years as children and below 5 years as infants. For the statistical analysis, we considered children as 1/2 of an adult, and infants as 1/3 of an adult [50]. On the same day, a co-author, not involved in the direct observations on ITN use, interviewed household heads about ITN use following the adult mosquito sampling (i.e., after 10 am). Since we found no major differences between interviews and direct observations (assuming direct observation as a gold standard, the sensitivity of the interviews was 0.93, the specificity was 0.85 and the kappa coefficient for agreement between the two methods was 0.69, for further details see Additional file 1: Supplement S1). Thus, given the intrusive nature of waking up householders to check their ITN use, during June and July, ITN use was assessed via interviews, and for the statistical analysis, we only employed the data from the interviews.

### Mosquito species identification, malaria infection diagnostic and bloodmeal identification

Sampled adult mosquitoes were killed in a freezer (−20°C) in Mbita after their collection. Anopheline mosquitoes were sorted into *An. gambiae s.l.* and *An. funestus s.l.* using the morphological criteria of Gillies and Coetzee [51]. Female mosquitoes were classified as fed, gravid, or unfed by examining their abdomen under a dissecting microscope. Chilled mosquitoes were transported to Nairobi, and upon arrival kept at −40°C to avoid the digestion of bloodmeals. In Nairobi (Nagasaki University laboratory at Kenya Medical Research Institute, KEMRI), mosquito samples were dissected transversely between the thorax and the abdomen, and legs were removed.

The head and thorax of each female mosquito were tested for malaria sporozoite infection using a *P. falciparum* circumsporozoite protein enzyme-linked immunosorbent assay (ELISA) [52]. DNA extracted from legs of each female mosquito [53] was employed for PCR based species identification within *An. gambiae s.l.*[54] and *An. funestus s.l.*[55]. Briefly, the PCR technique allows the identification of mosquitoes morphologically identified as *An.gambiae s.l.* into *An.gambiae s.s.* or *An. arabiensis*. Similarly, *An.funestus s.l.* mosquitoes can be separated into *An. funestus s.s., An rivulorum, An. leesoni* Evans*,* or *An. parensis* Gillies*.* Mosquitoes whose morphological and molecular identification was impossible were categorized as “other *Anopheles* mosquitoes”.

The abdomen of each blood-fed female mosquito was used for blood meal identification by ELISA and PCR. We employed these two techniques in order to use results from the most sensitive technique, i.e., the technique able to identify most of the bloodmeals in our mosquito samples, for the subsequent statistical analysis. Our ELISA was based in the protocol developed by Beier *et al*. [56]. Briefly, each female mosquito abdomen was homogenized in 1 ml of phosphate-buffered saline (PBS; pH7.4). Then, we identified blood meals, humans, cattle, goats/sheep, dogs or chicken, using IgG peroxidase antibodies (Sigma-Aldrich, Steinheim, Germany). For the tests, serum from a target species was employed as a positive control, while sera from the other species were negative controls. A reaction was positive when its absorbance was at least 2 times above the mean absorbance of the highest cross-reacting serum from hosts different from the target species.

For our PCR bloodmeal identification we also employed the PBS abdomen homogenate. From the homogenate we extracted DNA following the method described in Collins *et al*. [53]. This was also done to have positive controls for the multiplex PCR blood-meal identification protocol developed by Kent and Norris [57]. Briefly, we employed cytochrome-B primers for human (Human741F, ggcttacttctcttcattctctcct), cattle (Cow121F, catcggcacaaatttagtcg), dog (Dog368F, ggaattgtactattattcgcaaccat), a common primer for goat and sheep (Goat894F, cctaatcttagtacttgtacccttcctc) [58], and the universal reverse primer (UNREV1025, ggttgtcctccaattcatgtta) [57]. The PCR employed 10 ng of extracted DNA from the samples using an AccuPower™ Premix (Bioneer, Daejeon, Korea) under the following amplification conditions: 95°C for 5 min; 35 cycles of template denaturing at 95°C for 1 min, primer annealing at 54°C for 1 min, and amplicon extension at 72°C for 1 min; and a final extension at 72°C for 7 min. Each animal’s blood was detected by agarose gel electrophoresis (2% TAE) as 334 bp (Human) and 561 bp (Cow), 132 bp (Goat/Sheep), 680 bp (Dog) bands, respectively.

### Data analysis

We employed maximum likelihood statistical models to quantify the impacts of ITN use, presence of larval habitats, human and livestock abundance on: (i) *mosquito abundance*, (ii) *mosquito bloodfeeding on humans* and (iii) *mosquito sporozoite rate*. In each case the analysis began with a full model that considered all relevant covariates that we measured, which then was simplified until the Akaike Information Criterion (AIC) was minimized following the stepwise removal of covariates [59]. For (i) and (ii), we performed separate analyses for each dominant malaria vector species in the area, i.e., *An. arabiensis*, *An. gambiae s.s.* and *An. funestus s.s.,* and also for all the *Anopheles* spp. vectors combined. For (iii) we could only analyze *An. funestus s.s.* and the combination of all vector species we collected. All statistical analyses were performed with the Statistical Package R version 3.0, employing the library lme4 to fit generalized linear mixed effect models [60].

*Mosquito abundance*: for this analysis we employed Poisson generalized linear mixed models (Pois-GLMM). The response (i.e., independent variable) was the total number of mosquitoes, i.e., including gravid, unfed and bloodfed, collected by each PSC effort, i.e., per household and collection date. Fixed factors in the full model included: number of ITNs in use, distance to the closest permanent larval habitat from each household, abundance of ephemeral larval habitats in a given buffer area around each household, house area (as a proxy for adult mosquito resting habitat size), the month when each sample was collected (to control for seasonal mosquito abundance variability), the adjusted abundance of human residents in each household, cattle and goats/sheep in a given radius around each household, and number of neighboring households in a given radius around each household to account for the impact that humans living in the neighborhood could have in attracting mosquitoes to the focal house. Since some permanent larval habitats were too large to quantify in a given radius, we considered a distance from each household to the closest habitat as a proxy of their magnitude on potential adult mosquito productivity.

The radius, within which the abundance of ephemeral larval habitats, cattle, combined goats/sheep and neighboring households were counted, was chosen prior to the specification of the full model by a two step process. First we counted their abundance within a set of concentric circular areas with the following radii (with each radius originating from the center of each focal household): 20 m, 50 m, 100 m, 150 m, 200 m, 250 m, 300 m, 350 m, 400 m, 450 m, 500 m; and second we chose the best radius based on AIC minimization for Pois-GLMMs only considering mosquito abundance as a function of each factor in a given radius. We needed to perform this selection given the collinearity that emerges by considering the different radii simultaneously, since abundance in each radius is a linear function of a nested shorter radius, and collinear covariates can cause unidentifiability in parameter estimation by GLMMs [59]. The radii range was chosen based on the distances considered in previous zooprophylaxis studies in Russia [40], Pakistan [61], and western Kenya [34], to better account for the dispersal of anopheline mosquitoes, which on average is around 200 ~ 400 m [62]. Random factors included: the collection date to account for potential lack of temporal independence in our samples and the household identity to account for the potential lack of spatial independence [63].

*Human bloodfeeding*: for this analysis we employed binomial generalized linear mixed models (Bin-GLMMs). The response was the odds of mosquitoes having human bloodmeals versus bloodmeals from other hosts in each household and collection date. The odds of mosquitoes having human bloodmeals is defined as the ratio of the probability of mosquitoes having human bloodmeals divided by the probability of mosquitoes having a non-human bloodmeal. To compute the probability of human bloodmeals, we considered mosquitoes whose bloodmeal came exclusively from humans or humans and a second host. For the probability of a different host bloodmeal, we considered mosquitoes whose bloodmeal was identified as coming from a livestock species (cattle or goats/sheep) or was unidentified by the primers we employed. The full model included the same fixed and random factors used in the full Pois-GLMM used to study mosquito abundance.

*Mosquito sporozoite rate*: for this analysis we employed Poisson rate generalized linear mixed models (PoisR-GLMM). The response was the number of sporozoite infected mosquitoes. Briefly, the rate model considers the maximum number of sporozoite positive mosquitoes to be constrained by the number of collected mosquitoes that could have been infected (gravid or bloodfed mosquitoes are more likely infected than unfed mosquitoes) [64]. By contrast, a Pois-GLMM assumes this number as unconstrained [59]. As covariates we considered bednet coverage (i.e., the proportion of people in a household sleeping under an ITN) to control for differences in access to human hosts, the distance to permanent mosquito larval habitats, the abundance of ephemeral mosquito larval habitats in a given radius, house size and collection month. For the same reasons, we included these variables when analyzing *mosquito abundance*. We also considered cattle and goats/sheep blood indices, which are the proportion of mosquitoes with bloodmeals from each of these two hosts (excluding mixed meals with other hosts) divided by the total number of bloodfed mosquitoes. We employed the blood feeding indices as they allowed us to test if differential increases in the proportion of mosquitoes feeding on hosts other than humans decreases sporozoite infection (sporozoite rate of the vectors), or if other hosts provide a zooprophylactic effect.

## Results

### Mapping of livestock tethering points, mosquito larval habitats and ITN use

**Figure 3:**
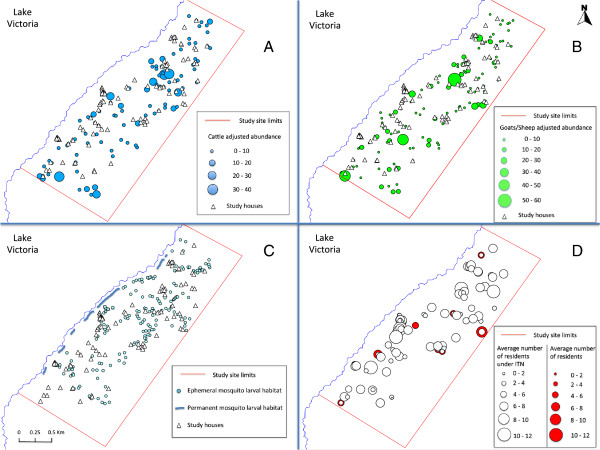
**Location, in relation to the study houses, of: tethering points for (A) Cattle (B) Goats and Sheep (C) *Anophele*s spp larval Habitats (D) Insecticide Treated Net (ITN) use.** For further details see the inset legends.

A total of 100 herds were present, comprising 850 cattle heads (adult: 746, calf: 104, adjusted abundance by size: 798.0) (Figure 3A). For goats/sheep, a total of 132 herds were present, and were comprised of 1301 goats (adult: 1094, young: 207, adjusted abundance by size: 1199.5), and 82 sheep (adult: 71, young: 11, adjusted abundance by size: 76.5) (Figure 3B).

All major permanent larval habitats (7 sites) with aquatic vegetation were located in Lake Victoria Coast, and a total of 173 ephemeral larval habitats were found (Figure 3C). Nearly 70% (118 sites) of the ephemeral habitats in the study area were natural pools such as puddles created in gullies. The remaining ones (55 sites) were mainly ditches or drainage channels associated with farms and pit holes.

Due to resident absence, of the 104 houses enrolled in the study, only 95 houses were surveyed in May, 100 in June and 100 in July. The average floor area of the houses was 17.37 ± 6.40 m^2^ (mean ± S.D.). The average number of people sleeping in a household was 4.19 ± 1.95 (mean ± S.D.), with minimum variation across the months of our study (Figure 3D). The average number of ITNs in use per house was 1.48 ± 0.63 (mean ± S.D.) with non-significant variation through our study, and the average number of residents sleeping under ITNs per house was 3.47 ± 1.90 (mean ± S.D.). Throughout our study, an average of 82% of the people sleeping in our study houses used ITNs. There were some heterogeneities regarding the use by age group. About 90% of the adults were covered, but only 65% of the children and 83% of the infants slept under ITNs.

### Mosquitoes, bloodmeal hosts and sporozoite rate

**Figure 4:**
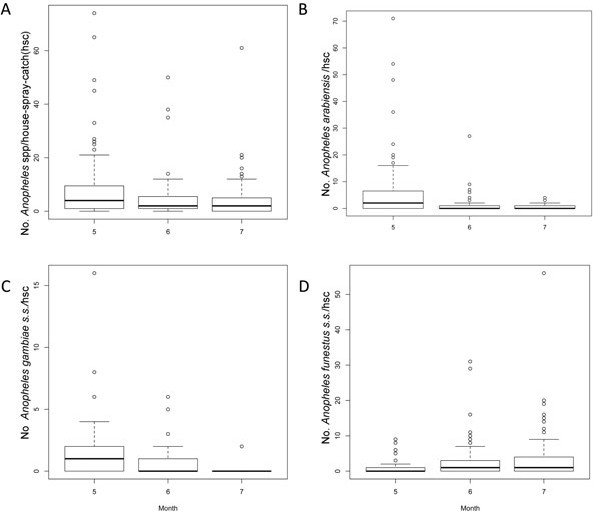
**Mosquito Abundance per household spray catch and month (A) All *Anopheles *** **spp (B) *Anopheles arabiensis *** **(C) *Anopheles gambiae s.s. *** **(D) *Anopheles funestus s.s..***

In total we collected 8123 mosquitoes over 295 sessions of PSC, and 1664 of them were female mosquitoes belonging to either *An. gambiae s.l*. or *An. funestus s.l.* (811 in May, 416 in June, and 437 in July). According to the morphological species identification, 928 *Anopheles* mosquitoes were *An. gambiae s.l.*, which, using PCR were further identified as: 726 *An. arabiensis*, 196 *An. gambiae s.s..* Belonging to *An. funestus s.l.,* we morphologically identified 736 individuals, with 711 being *An. funestus s.s.*, and 1 *An. rivulorum*. We could not molecularly identify 6 *An. gambiae s.l.* and 24 *An. funestus s.l.* with the PCR method. The abundance of *Anopheles* vectors was nearly constant during the study period (Figure 4A). *Anopheles arabiensis* was mainly present in the early months of our study (Figure 4B), and *An. funestus s.s.* in July (Figure 4D). Meanwhile, *An. gambiae s.s.* had its maximum abundance in May, almost disappearing in July (Figure 4C).

We tried to identify host bloodmeals in all the 1204 fed *Anopheles* vectors, using the Kent & Norris multiplex PCR (Additional file 2: Table S1) and ELISA (Additional file 3: Table S2). With the PCR method we identified bloodmeal hosts for 1031 (85.63%) samples, while the ELISA only allowed the identification of blood sources in 846 (70.27%) samples. Thus, the rest of our results and analysis will be exclusively based on the PCR data. Main vectors where blood meals were identified were: *An. arabiensis* (n = 440), *An. gambiae s.s.* (n = 110), *An. funestus s.s.* (n = 461). Most bloodmeals for *An. gambiae s.s.* and *An.funestus s.s.* came from humans (See Additional file 2: Table S1). The proportions of human bloodmeal for *An. gambiae s.s.*, *An. funestus s.s.* and *An. arabiensis* were 49.5% (CI: 42.3-56.7), 63.9% (CI: 60.2-67.4) and 12.0% (CI: 9.7-14.6), respectively, and the proportion for *An. arabiensis* was significantly less compared with the other species. Meanwhile, the major blood source was cattle for *An. arabiensis.* The proportions of cattle bloodmeal for *An. gambiae s.s.*, *An. funestus s.s.* and *An. arabiensis* were 1.2% (CI: 0.6-2.3), 2.0% (CI: 0.6-5.1) and 40.5% (CI: 36.9-44.2), respectively, and the proportion for *An. arabiensis* was significantly greater.

Two *An. arabiensis*, three *An.gambiae s.s.* and 21 *An. funestus s.s.* had *P. falciparum* sporozoites. Sporozoite rate was 0.28% (CI: 0.03-0.99) for *An. arabiensis*, 1.53% (CI: 0.32-4.40) for *An. gambiae s.s.* and 2.95% (CI: 1.83-4.48) for *An. funestus* s.s., and the rate for *An. funestus s.s.* was significantly higher compared with *An. arabiensis*.

### Statistical models

For all models we indicated the number of observations in the different tables. We ignored factors that showed no statistical significance (P > 0.05) in the description of the best models results, since, in principle, the impacts of those factors are not different from what is expected by random. Here, we also want to mention that for some models spatial or temporal variability was not significant, thus explaining their absence in the final models and during the process of model selection. All the assumptions of the statistical models were not violated.

#### The impacts of ITN use, presence of larval habitats, human and livestock abundance on mosquito abundance

After a process of model selection (Additional file 4: Table S3), we found that the best model explaining the abundance of all the *Anopheles* vectors included ITN use, goats/sheep abundance in 20 m around a household, and the adjusted number of residents in a household (Table 1). We found the number of mosquitoes per PSC session, increased 9% by each additional goat or sheep tethered within a 20 m radius from a household, and 36% per each additional person sleeping in a household. By contrast mosquito abundance was halved by each ITN use. We also found a higher spatial variability (Household variance in Table 1) than temporal variability (Collection date variance in Table 1).

**Table 1:** **Parameter estimates for the best Poisson GLMM explaining the abundance of *Anopheles *** **vectors**

**Parameter**	**Exp (β)**	**β**	**SE**	**Z**	**P**
Intercept	-	0.79	0.34	2.32	**0.02**
Goats/sheep in 20 m	1.09	0.09	0.03	2.64	**0.01**
ITNs in use	0.48	−0.73	0.08	−9.07	**< 0.01**
Residents	1.36	0.30	0.04	6.77	**< 0.01**
House size	1.03	0.03	0.02	1.68	0.09
Household variance	-	0.85	-	-	-
Date variance	-	0.37	-	-	-

All vector species were combined in this analysis.

P values in bold are statistically significant (P < 0.05).

We also performed a process of model selection for each one of the dominant vector species (Additional file 5: Table S4). We found that *An. arabiensis* abundance was increased by 10% with each additional goats/sheep tethered within 20 m of a household, 6% by each square meter of household basal area, that its abundance increased around 10 times in May when compared with July, while ITNs nearly halved its abundance, and each neighbouring household within a 50 m radius decreased mosquito abundance by 5% (Table 2). The impacts of ITNs on *An. gambiae s.s.* abundance were stronger. For each ITN, mosquito abundance decreased by 58%, its abundance also decreased with the number of neighbouring houses, and it increased by 36% per each additional household resident (Table 2). Further, the abundance was increased by 92% in May when compared with July. As observed in the raw data, *An. funestus s.s.* abundance was maximum in July. Each additional resident increased its abundance by 68%, while each ITN in use reduced it by 59% (Table 2). Interestingly, *An. funestus s.s.* abundance increased by 3% with each additional ephemeral habitat in a 500 m radius around the house. For all the dominant vector species, spatial variability (Household variance in Table 2) was larger than temporal variability (Date variance in Table 2).

**Table 2:** Parameter estimates for the best Poisson GLMM explaining the abundance of three vector species

	***An. arabiensis *** **(n = 295)**					***An. gambiae s.s. *** **(n = 195)**					***An. funestus s.s*. (n = 295)**				
**Parameter**	**Exp (β)**	**β**	**SE**	**Z**	**P**	**Exp (β)**	**β**	**SE**	**Z**	**P**	**Exp (β)**	**β**	**SE**	**Z**	**P**
Intercept	-	−1.53	0.66	−2.33	**0.02**	-	−0.06	0.35	−0.16	0.87	-	−0.43	0.54	−0.80	0.43
Goats/sheep in 20 m	1.10	0.10	0.04	2.43	**0.02**	-	-	-	-	-	-	-	-	-	-
ITNs in use	0.54	−0.62	0.14	−4.55	**< 0.01**	0.42	−0.87	0.15	−5.90	**< 0.01**	0.41	−0.88	0.13	−6.77	**< 0.01**
Residents	-	-	-	-	-	1.36	0.31	0.09	3.40	**< 0.01**	1.68	0.52	0.07	7.25	**< 0.01**
Houses in 50 m	0.95	−0.05	0.02	−2.25	**0.02**	0.94	−0.06	0.02	−3.44	**< 0.01**	-	-	-	-	-
Houses in 150 m	-	-	-	-	-	-	-	-	-	-	1.02	0.02	0.01	1.59	0.11
Ephemeral habitats in 500 m	-	-	-	-	-	-	-	-	-	-	1.03	0.03	0.01	2.31	**0.02**
Month (May)	9.87	2.29	0.53	4.36	**< 0.01**	1.92	0.65	0.22	3.00	**< 0.01**	0.22	−1.53	0.39	−3.90	**< 0.01**
Month (June)	2.13	0.75	0.52	1.45	0.15	-	-	-	-	-	0.56	−0.57	0.37	−1.56	0.12
House size	1.06	0.06	0.02	2.36	**0.02**	-	-	-	-	-	-	-	-	-	-
Household variance	-	1.16	-	-	-	-	0.39	-	-	-	-	1.34	-	-	-
Date variance	-	0.59	-	-	-	-	0.05	-	-	-	-	0.31	-	-	-

We only considered May and June samples for *An. gambiae s.s*., because few were collected in July.

P values in bold are statistically significant (P < 0.05).

#### The impacts of ITN use, presence of larval habitats, human and livestock abundance on mosquito human bloodfeeding

Model selection (Additional file 6: Table S5) showed that when all *Anopheles* vector species were combined, bloodfeeding on humans was significantly associated with the number of people sleeping in a household, which increased the odds of an *Anopheles* mosquito feeding on humans by 1.53 times, and odds of human bloodfeeding were decreased 0.99 times by each goat or sheep tethered within 500 m from the household (Table 3). By contrast odds of human bloodfeeding in May was decreased 0.20 times when compared with July, and additional square meter of household basal area decreased it 0.96 times.

**Table 3:** **Parameter estimates for the best binomial GLMM explaining feeding on humans by *Anopheles *** **vectors**

**Parameter**	**Exp (β)**	**β**	**SE**	**Z**	**P**
Intercept	-	1.51	0.62	2.46	**0.01**
Goats/Sheep in 500 m	0.99	−0.01	2.E-03	−3.01	**< 0.01**
ITNs in use	0.77	−0.26	0.16	−1.61	0.11
Residents	1.53	0.42	0.09	4.52	**< 0.01**
Houses in 50 m	1.04	0.04	0.02	1.82	0.07
Month (May)	0.20	−1.61	0.47	−3.43	**< 0.01**
Month (June)	1.11	0.10	0.47	0.22	0.83
House size	0.96	−0.04	0.02	−2.44	**0.01**
Household variance	-	0.27	-	-	-
Date variance	-	0.43	-	-	-

All vector species were combined in this analysis.

P values in bold are statistically significant (P < 0.05).

N = 208.

Regarding each dominant vector species in the area, we found that factors influencing human bloodfeeding were different (Additional file 7: Table S6). We found that *An. gambiae s.s.* was significantly sensitive only to the presence of humans, where each additional human increased human bloodfeeding odds two times (Table 4). A similar pattern was also observed for *An. funestus s.s..* Its human bloodfeeding significantly increased 1.4 times with each additional person sleeping in a house, and was 4.4 times more likely to happen in June (Table 4). By contrast, human bloodfeeding in *An. funestus s.s.* decreased 0.93 times with each additional ephemeral habitat in a 200 m radius around a house. While human bloodfeeding in *An. arabienesis* was 1.54 times most likely by the presence of each human in the household, it also increased 1.01 times for each goats/sheep tethered within 500 m from a household and decreased with the number of cattle heads tethered in the same radius as the goats/sheep (Table 4). *Anopheles arabiensis* feeding on humans was 1.07 times more likely to occur as the number of houses in 150 m around a focal household increased and human bloodfeeding was 0.69 times less likely to occur during May compared with July. The temporal variances for *An. arabiensis* and *An. gambiae s.s.* were around 0.30 (Date variance in Table 4) which, respectively, represent 0.23% and 0.36% of the total deviance (*An. arabiensis* deviance = 129.7 and *An gambiae s.s.* deviance =83.1). The spatial variance for *An. funestus s.s.* was very low (Household variance in Table 4) representing only 0.25% of the total deviance (*An funestus s.s.* deviance =121.0).

**Table 4:** Parameter estimates for the best Binomial GLMM explaining feeding on humans by three vector species

	***An.arabiensis *** **(n = 109)**					***An.gambiae s.s. *** **(n = 63)**					***An.funestus s.s. *** **(n = 129)**				
**Parameter**	**Exp (β)**	**β**	**SE**	**Z**	**P**	**Exp (β)**	**β**	**SE**	**Z**	**P**	**Exp (β)**	**β**	**SE**	**Z**	**P**
Intercept	-	−2.31	0.74	−3.11	**< 0.01**	-	−0.30	0.64	−0.46	0.64	-	1.76	0.47	3.71	**< 0.01**
Cattle in 50 m	-	-	-	-	-	-	-	-	-	-	0.92	−0.08	0.05	−1.73	0.08
Cattle in 500 m	0.98	−0.02	0.01	−3.03	**< 0.01**	-	-	-	-	-	-	-	-	-	-
Goats/Sheep in 20 m	-	-	-	-	-	0.88	−0.12	0.07	−1.78	0.08	-	-	-	-	-
Goats/Sheep in 300 m	-	-	-	-	-	-	-	-	-	-	0.99	−0.01	4.E-03	−1.61	0.11
Goats/Sheep in 500 m	1.01	0.01	0.01	2.25	**0.02**	-	-	-	-	-	-	-	-	-	-
ITNs in use	-	-	-	-	-	0.57	−0.57	0.33	−1.70	0.09	0.74	−0.31	0.22	−1.42	0.16
Residents	1.54	0.43	0.12	3.52	**< 0.01**	2.02	0.70	0.22	3.22	**< 0.01**	1.40	0.33	0.12	2.68	**0.01**
Houses in 150 m	1.07	0.06	0.01	4.26	**< 0.01**	-	-	-	-	-	-	-	-	-	-
Ephemeral habitats in 200 m	-	-	-	-	-	-	-	-	-	-	0.93	−0.08	0.04	−2.06	**0.04**
Month (May)	0.31	−1.16	0.54	−2.16	**0.03**	-	-	-	-	-	0.97	−0.03	0.35	−0.10	0.92
Month (June)	0.72	−0.32	0.58	−0.56	0.58	-	-	-	-	-	4.43	1.49	0.42	3.55	**< 0.01**
Household variance	-	-	-	-	-	-	-	-	-	-	-	7.E-13	-	-	-
Date variance	-	0.31	-	-	-	-	0.32	-	-	-	-	-	-	-	-

P values in bold are statistically significant (P < 0.05).

#### The impact of ITN use, presence of larval habitats, human and livestock abundance on mosquito sporozoite rates

The sporozoite rate in all *Anopheles* vector species combined was significantly associated with the number of ephemeral habitats in 250 m radius around the houses, where each habitat increased the sporozoite rate by 13%. In contrast the rate reduced by 9.2% as the proportion of mosquitoes feeding on cattle increased by 10% (Table 5, for variable selection see Additional file 8: Table S7). Regarding *An. funestus s.s.,* the species with the largest number of sporozoite positive individuals, we found that sporozoite rate increased by 15% with each ephemeral habitat in a 250 m radius around the house (Table 5, for variable selection see Additional file 9: Table S8). The random factors had a very low variability, less than 0.1% of the total deviance (Table 5).

**Table 5:** Parameter estimates for Poisson rate GLMM explaining the sporozoite rate of malaria vectors

	***Anopheles *** **spp. (n = 208)**					***An.funestus s.s. *** **(n = 129)**				
**Parameter**	**Exp (β)**	**β**	**SE**	**Z**	**P**	**Exp (β)**	**β**	**SE**	**Z**	**P**
Intercept	-	−4.66	0.55	−8.53	**< 0.01**	-	−4.75	0.67	−7.05	**< 0.01**
Cattle blood index	0.08	−2.58	0.94	−2.75	**0.01**	-	-	-	-	-
Ephemeral habitats in 250 m	1.13	0.12	0.05	2.40	**0.02**	1.15	0.14	0.06	2.26	**0.02**
Household variance	-	2.E-12	-	-	-	-	-	-	-	-
Date variance	-	6.E-14	-	-	-	-	6.E-20	-	-	-

All vector species were combined in this analysis for *Anopheles* spp..

P values in bold are statistically significant (P < 0.05).

## Discussion

The role of zooprophylaxis, more generally of bloodmeal source diversity, on vector-borne disease has been controversial since early times [32,37,47,65,66]. An outstanding conclusion from field studies [32,47,48] and mathematical modeling [38,39,67,68] is that diversion of feeding to alternative hosts needs to outweigh the potential increase in mosquito population size and attraction to humans by “zooprophylactic” animals [31]. To be more specific, mosquitoes may increase their contact with humans after they are attracted to the proximity of a household, or mosquito population size may increase, because of increases in livestock presence and hoofprint habitats for mosquito larvae [69,70]. All these factors seem to critically rely on the location and abundance of potential alternative hosts.

Nevertheless, previous field studies with negative results for zooprophylaxis neglected the exact location of overnight tethering points for livestock. They mainly looked at heterogeneities in livestock ownership via interviews [47,48,61]. Moreover, the information gathered by the interviews may provide imprecise information about alternative host availability, for example, the cattle belonging to a household is not necessarily the one tethered close to it. To overcome this limitation, we performed a census of all livestock present in our study area, georeferencing overnight tethering points, herd size and the age structure of herds.

### Zooprophylaxis in the community of Malaria vector species

Unlike a previous comprehensive study on passive zooprophylaxis in West Africa [47,48], we found that increases in bloodfeeding in cattle can reduce the likelihood of vector infection, i.e., they might render a zooprophylactic effect. Thus, our data suggest, that for the overall community of malaria vectors, cattle presence might be acting as a “pulling” passive malaria control strategy in areas where high ITN coverage have “pushed” changes in mosquito access to humans.

In our study area, although the abundance of goats/sheep tethered in close proximity (20 m or less) to a household is associated with an increase in the number of *Anopheles* mosquitoes that can be caught in such house, a phenomenon already described [8], the main driver of domiciliary mosquito abundance seems to be the number of people sleeping in a house. Nevertheless, it is well documented that people sleeping under ITNs have a significant reduction in their malaria infection risk [71], because, as shown by this study and others, the number of mosquitoes is significantly smaller in houses where people use ITNs [7,72]. In fact, our parameter estimates suggest that ITN use can outweigh the increase in mosquito density by the presence of one human and up to 4 goats/sheep. For example, if we estimate the proportional change in mosquito abundance as a function of these factors ((1.09)^4^ × 1.36 × 0.48 = 0.92, Table 1), we still can expect an 8% reduction in mosquito abundance because of ITN use. If we consider the subset of bloodfed *Anopheles* mosquitoes, we also see that odds of bloodfeeding on humans are primarily associated with human abundance and ITN use. Similar phenomena have been observed in previous studies about bloodfeeding in African settings with ITNs [14,73].

**Figure 5:**
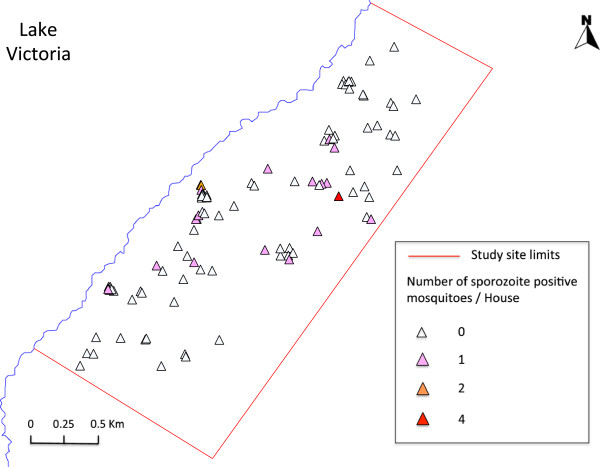
**House location and abundance of sporozoite positive *Anopheles *spp mosquitoes.** For further details see the inset legend.

Nevertheless, the likelihood of vector infection can also be increased by the presence of ephemeral habitats. Since most of the malaria infected mosquitoes were *An. funestus s.s.*, whose malaria infection was only increased in the presence of ephemeral habitats, the association of ephemeral habitats can be related to two factors. The first is that, *An. funestus s.s.,* whose immature stages mainly develop in permanent swamps along Lake Victoria shore [42], may be blown into the area where infected mosquitoes were found. We have observed that *Anopheles* mosquitoes emerge in the evening when the west wind from the lake is strong, and then given the small body size and relative poor flight ability of this vector species, they may get stuck in the area. In fact, several infected mosquitoes were found in houses east of two large permanent habitats (Figure 3C and Figure 5). This explanation may also be applied to the association of *An. funestus s.s.* abundance with ephemeral habitats that were also recorded east of the permanent habitats (Figure 3C).

The second factor, although uncommon, *An. funestus s.s.* larvae can colonize what we classified as ephemeral habitats [74], probably reflects some sub-optimal oviposition habitat selection, a phenomenon commonly observed in mosquitoes [75,76]. Furthermore, the suboptimal oviposition might be also related to the limited dispersal ability of *An. funestus s.s.* mosquitoes and the fitness costs of not ovipositing when it is preferable to do so in a suboptimal habitat and bloodfeed again. This can increase malaria transmission [67,68] and might be common, since it has been observed in other mosquito vectors [77]. Although this explanation seems counterintuitive given that human bloodfeeding in *An. funestus s.s.*, slightly decreased with an increase of ephemeral habitats in a 200 m radius, infection still can happen as long as mosquitoes bloodfeed on infected humans.

### Zooprophylaxis on the dominant malaria vectors

Regarding zooprophylactic patterns in each dominant vector species, we found that there were species-specific differences. *Anopheles gambiae s.s.*, the less common species after ITN coverage has been scaled in the study area [7], had abundance patterns that were insensitive to livestock abundance, and its feeding on humans decreased (yet not significantly) in the presence of goats and sheep [78]. Nevertheless, its household abundance greatly decreased in the presence of ITNs, and the number of sporozoite positive *An. gambiae s.s*. was only three, meaning that ITNs might be controlling malaria transmission by this species, as suggested by previous studies [7].

Although not statistically significant in the best model selected by AIC, the presence of cattle and goats/sheep reduced the odds of human bloodfeeding in *An. funestus s.s*. by magnitudes (8% and 1%, respectively) that prevail over the potential positive impacts of cattle on malaria transmission. This species accounted for most of the sporozoite infected mosquitoes and infections might reflect differential patterns of human exposure to this species because of human activities.

*Anopheles arabiensis*, showed the most complex relationships with livestock abundance, first it increased about 10% for each goat or sheep tethered in close proximity (20 m or less) to the household. This species also increased its bloodfeeding on humans with the overall numbers of goats/sheep in the landscape (500 m or less). Goats and sheep often spend time outside house walls under extended eaves. They may release semiochemicals, which could be associated with the “goat” fragrance familiar to anybody working with goats and sheep, and goat associated semiochemicals could potentially attract this vector close to the houses. Nevertheless, its feeding on humans decreased with cattle abundance in the landscape (500 m or less), which had no impact on its abundance. The positive impacts of cattle might reflect the low number of mosquitoes with malaria sporozoites. In synthesis, cattle seem to provide a zooprophylactic effect in *An. arabiensis* and *An. funestus s.s.*. Goats/sheep abundance is nevertheless associated with an increase in *An. arabiensis* abundance and likelihood of feeding on humans.

### Study limitations

The limited span of our study can only provide inferences about the rainy season. Ideally, a longer study would provide a better picture of the magnitude of zooprophylactic effects by different livestock species on the community of malaria vectors year-round. The increased sampling could also improve the power of our estimates, for example, telling if the non-significance of factors in the models selected as best were artifacts of the sample size, and to better understand any impacts of weather seasonality on feeding behavior and abundance of household resting mosquitoes [79].

Our sampling method only focused on collecting indoor resting mosquitoes by PSC without collecting mosquitoes outside households. We could have underestimated the number of mosquitoes, especially the species that have a preference to feed indoors (endophagy) on humans (anthropophagy), but mainly rest outdoors (exophily) [17]. Moreover, our sampling method might totally ignore exophilic and exophagic species. Many of those exo-exo mosquito species are vectorially competent (i.e., get infected and develop sporozoites) for human malaria parasites and might be responsible for some malaria transmission in the area.

We could also have collected additional data on income, similar to previous studies [48], or measure specific details about house conditions such as eave openness, which affect malaria vector household entrance behavior [80]; that is, we limited our inferences to a relatively homogenous group of households.

### Perspectives and recommendations for future studies

Beyond improving the limitations of our study, we believe there is also room to explore the origin of unidentified bloodmeals, and the impact of the bloodmeal source hosts in zooprophylaxis. For example, Kawada *et al*. [17] found *An. rivolorum* feeding on hippopotamus, a common vertebrate in Lake Victoria, suggesting that malaria vectors can have unappreciated hosts [78]. These hosts may contribute to zooprophylaxis, and the impacts of unappreciated hosts could be related to their diversity, a hypothesis originally envisioned by Celli when talking about zooprophylaxis [32,36] and refereed as “dilution effect” by some ecologists working with ticks, who have seen decreases in disease risk/burden when biodiversity increases [32,65,66]. Zooprophylaxis impacts can also be potentially enhanced by the use of livestock vermicides [81,82], which can kill mosquitoes after bloodfeeding on livestock hosts. In addition, it will be worth exploring the use of insecticide collars [83-85] in goats tethered around houses, which potentially can further “push” malaria vectors out of human contact.

## Conclusion

Our data suggest that malaria control by high ITN coverage might be benefiting from zooprophylactic effects derived from the abundance of cattle in our study area. The synergy between zooprophylaxis and ITNs resulted in an integrated vector control strategy for the dominant vector species in our study area, especially for *An. arabiensis*. This integrated “push-pull” strategy may also be effective for reducing malaria transmission in the other parts of LVB and similar sites where *An. arabiensis* is a dominant vector species. Our data also suggest that tethering and keeping goats as far as possible from households could improve zooprophylaxis in *An. arabiensis*, whose abundance and feeding on humans increases with the density of goats tethered in close proximity to households.

## Competing interests

All authors declare that they have no competing interests.

## Authors’ contributions

HI and NM conceived and designed this study. SN helped designing and planning the study in Kenya. GD, GS, and HI collected the field data, and HI and KF organized and conducted the laboratory work. HI, TS, LFC and NM performed the data analyses. HI drafted the first manuscript, and LFC and NM finalized the manuscript. All authors have read and approved the final manuscript.

## Supplementary Material

Additional file 1: Supplement S1 Comparison of direct observation vs interview to estimate insecticide treated net (ITN) use. Click here for file

Additional file 2: Table S1 Female *Anopheles* spp. mosquito abundance by month, parity (gravid), feeding status and PCR based bloodmeal source. Click here for file

Additional file 3: Table S2 ELISA based bloodmeal sources in fed *Anopheles* spp. mosquitoes. Click here for file

Additional file 4: Table S3 Poisson GLMM selection for the abundance of all *Anopheles* species combined. Click here for file

Additional file 5: Table S4 Poisson GLMM selection for the abundance of each *Anopheles* species. Click here for file

Additional file 6: Table S5 Binomial GLMM selection for *Anopheles* spp. mosquito feeding on humans over other bloodmeal sources. Click here for file

Additional file 7: TableS 6 Binomial GLMM selection for each *Anopheles* species feeding on humans over other bloodmeal sources. Click here for file

Additional file 8: Table S7 Poisson rate GLMM selection for the sporozoite rate of all *Anopheles* spp. combined. Click here for file

Additional file 9: Table S8 Poisson rate GLMM selection for the sporozoite rate of *Anopheles funestus s.s..* Click here for file
