# Supplementary material for: Push by a net, pull by a cow: can zooprophylaxis enhance the impact of insecticide treated bed nets on malaria control?
Source: Parasit Vectors. 2014 Jan 28;7:52. doi: 10.1186/1756-3305-7-52 (PMC3917899; doi:10.1186/1756-3305-7-52)
Supplement: Additional file 1: Supplement S1 — Comparison of direct observation vs interview to estimate insecticide treated net (ITN) use. [file 1756-3305-7-52-S1.docx]

**Supplement S1.** Comparison of direct observation vs interview to estimate insecticide treated net (ITN) use.

Direct observation can be assumed as a gold standard (i.e., the most accurate method) for measuring ITN use. Nevertheless, it is an intrusive method and it is not clear how different are the results obtained with this method when compared with those obtained from interviews to household heads. Thus, in this supplement, we briefly explain how did we compare the two methods and why did we decide to only use interviews during the whole study. First, we estimated the sensitivity and specificity of the interviews to measure ITN use, employing the results from direct observation of ITN use as a gold standard. In the context of our study sensitivity is the ability to correctly estimate the number of people that used ITNs, while specificity is the ability to correctly estimate the number of people that did not use ITNs. For the estimation of interview based ITN use sensitivity and specificity we employed the cross classified data from the two methods:

| Cross-classification | | Interview | |
| --- | --- | --- | --- |
|  |  | Yes | No |
| Direct  Observation | Yes | 236 | 6 |
|  | No | 17 | 35 |

Where rows indicate the results for the direct observation and columns for the interviews, and yes indicates the number of people that slept under the ITNs, while No the ones that didn’t use ITNs. Sensitivity is the ratio obtained when the true positives, i.e., yes-yes in the table, are divided by the number of positives by the interview method [[1](#_ENREF_1)]. Similarly, specificity is the ratio obtained when the true negatives, i.e., no-no in the table, are divided by the number of negatives by the interview method [[1](#_ENREF_1)]. In general sensitivities of 0.95 or higher are considered desirable, while specificities above 0.80 indicate a highly specific classification [[1](#_ENREF_1)].

For robustness, we also estimated Cohen’s kappa coefficient for the two methods, which is a measurement of the agreement between two methods [[2](#_ENREF_2)]. The kappa coefficient is estimated by the following expression:

$$kappa=\frac{\Pr\left( a \right)-Pr(e)}{1-Pr(e)}$$

where Pr(a) is the probability of agreement between the two methods, and Pr(e) the probability of agreement arising by chance. The original article by Cohen [2] succinctly explains the estimation of Pr(a) and Pr(e) from the cross-classification table. According to Landis and Koch [[3](#_ENREF_3)] an agreement above 0.60 is substantial, meaning the outcome by both methods is very similar.

Thus, given the high specificity and sensitivity of the interviews, as well as, the substantial agreement between the two methods we decided to only employ the interviews through the whole study.

**References**

1. Altman DG, Bland JM: **Statistics Notes: Diagnostic tests 1: sensitivity and specificity**. *BMJ* 1994, **308**(6943):1552.

2. Cohen J: **A Coefficient of Agreement for Nominal Scales**. *Educational and Psychological Measurement* 1960, **20**(1):37-46.

3. Landis JR, Koch GG: **The measurement of observer agreement for categorical data**. *Biometrics* 1977:159-174.
