# Supplementary material for: Push by a net, pull by a cow: can zooprophylaxis enhance the impact of insecticide treated bed nets on malaria control?
Source: Parasit Vectors. 2014 Jan 28;7:52. doi: 10.1186/1756-3305-7-52 (PMC3917899; doi:10.1186/1756-3305-7-52)
Supplement: Additional file 2: Table S1 — Female Anopheles spp. mosquito abundance by month, parity (gravid), feeding status and PCR based bloodmeal source. [file 1756-3305-7-52-S2.docx]

| Table S1. Female *Anopheles* spp. mosquito abundance by month, parity (gravid), feeding status and PCR based bloodmeal source. | | | | | | | | | | | | |
| --- | --- | --- | --- | --- | --- | --- | --- | --- | --- | --- | --- | --- |
| Species | Collection month | gravid | unfed | Blood fed mosquitoes | | | | | | | | Total |
|  |  |  |  | Cattle only | Goat (Sheep) only | Dog only | Human only | Human/  Cattle | Human/  Goat (Sheep) | Cattle/  Goat (Sheep) | unknown |  |
| *An. arabiensis* | May | 43 | 157 | 225 | 18 | 10 | 56 | 17 | 2 | 4 | 44 | 576 |
|  | June | 12 | 9 | 42 | 0 | 0 | 20 | 2 | 0 | 2 | 15 | 102 |
|  | July | 2 | 2 | 21 | 4 | 1 | 11 | 4 | 0 | 1 | 2 | 48 |
| *An. gambiae s.s.* | May | 11 | 31 | 8 | 3 | 1 | 65 | 0 | 0 | 0 | 15 | 134 |
|  | June | 14 | 5 | 1 | 0 | 0 | 30 | 0 | 0 | 0 | 10 | 60 |
|  | July | 0 | 0 | 0 | 0 | 0 | 2 | 0 | 0 | 0 | 0 | 2 |
| Other *An. gambiae s.l.* group | May | 0 | 0 | 0 | 0 | 0 | 1 | 0 | 0 | 0 | 0 | 1 |
|  | June | 1 | 0 | 1 | 0 | 0 | 2 | 0 | 0 | 0 | 0 | 4 |
|  | July | 0 | 0 | 0 | 1 | 0 | 0 | 0 | 0 | 0 | 0 | 1 |
| *An. funestus s.s.* | May | 13 | 18 | 0 | 0 | 1 | 53 | 1 | 0 | 0 | 12 | 98 |
|  | June | 65 | 23 | 0 | 0 | 0 | 146 | 0 | 0 | 0 | 7 | 241 |
|  | July | 22 | 25 | 4 | 0 | 0 | 255 | 1 | 0 | 0 | 65 | 372 |
| *An. rivulorum* | May | 0 | 0 | 0 | 0 | 0 | 0 | 0 | 0 | 0 | 0 | 0 |
|  | June | 0 | 0 | 1 | 0 | 0 | 0 | 0 | 0 | 0 | 0 | 1 |
|  | July | 0 | 0 | 0 | 0 | 0 | 0 | 0 | 0 | 0 | 0 | 0 |
| Other *An. funestus s.l.* group | May | 0 | 0 | 0 | 0 | 0 | 2 | 0 | 0 | 0 | 0 | 2 |
|  | June | 4 | 1 | 0 | 0 | 0 | 3 | 0 | 0 | 0 | 0 | 8 |
|  | July | 2 | 0 | 1 | 0 | 0 | 8 | 0 | 0 | 0 | 3 | 14 |
| Total |  | 189 | 271 | 304 | 26 | 13 | 654 | 25 | 2 | 7 | 173 | 1664 |
