# Supplementary material for: Push by a net, pull by a cow: can zooprophylaxis enhance the impact of insecticide treated bed nets on malaria control?
Source: Parasit Vectors. 2014 Jan 28;7:52. doi: 10.1186/1756-3305-7-52 (PMC3917899; doi:10.1186/1756-3305-7-52)
Supplement: Additional file 3: Table S2 — ELISA based bloodmeal sources in fed Anopheles spp. mosquitoes. [file 1756-3305-7-52-S3.docx]

| Table S2. ELISA based bloodmeal sources in fed *Anopheles* spp. mosquitoes. | | | | | | | | | | | |
| --- | --- | --- | --- | --- | --- | --- | --- | --- | --- | --- | --- |
| Species | Collection month | Cattle only | Goat (Sheep) only | Dog only | Human only | Bovine/  Goat (Sheep) | Cattle/Dog | Chicken/Human | Cattle/  Goat (Sheep)  /Dog | unknown | Total |
| *An. arabiensis* | May | 211 | 11 | 15 | 49 | 10 | 1 | 0 | 1 | 78 | 376 |
|  | June | 42 | 1 | 1 | 19 | 5 | 0 | 0 | 0 | 13 | 81 |
|  | July | 22 | 3 | 1 | 4 | 3 | 0 | 0 | 0 | 11 | 44 |
| *An. gambiae s.s.* | May | 6 | 1 | 1 | 45 | 1 | 0 | 1 | 0 | 37 | 92 |
|  | June | 2 | 0 | 0 | 25 | 0 | 0 | 0 | 0 | 14 | 41 |
|  | July | 0 | 0 | 0 | 2 | 0 | 0 | 0 | 0 | 0 | 2 |
| Other *An. gambiae s.l.* group | May | 0 | 0 | 0 | 1 | 0 | 0 | 0 | 0 | 0 | 1 |
|  | June | 1 | 0 | 0 | 2 | 0 | 0 | 0 | 0 | 0 | 3 |
|  | July | 0 | 0 | 0 | 0 | 0 | 0 | 0 | 0 | 1 | 1 |
| *An. funestus s.s.* | May | 0 | 0 | 1 | 46 | 0 | 0 | 0 | 0 | 20 | 67 |
|  | June | 0 | 0 | 0 | 106 | 0 | 0 | 0 | 0 | 47 | 153 |
|  | July | 3 | 0 | 0 | 195 | 0 | 0 | 0 | 0 | 127 | 325 |
| *An. rivulorum* | May | 0 | 0 | 0 | 0 | 0 | 0 | 0 | 0 | 0 | 0 |
|  | June | 1 | 0 | 0 | 0 | 0 | 0 | 0 | 0 | 0 | 1 |
|  | July | 0 | 0 | 0 | 0 | 0 | 0 | 0 | 0 | 0 | 0 |
| Other *An. funestus s.l.* group | May | 0 | 0 | 0 | 2 | 0 | 0 | 0 | 0 | 0 | 2 |
|  | June | 0 | 0 | 0 | 1 | 0 | 0 | 0 | 0 | 2 | 3 |
|  | July | 0 | 0 | 0 | 4 | 0 | 0 | 0 | 0 | 8 | 12 |
| Total |  | 288 | 16 | 19 | 501 | 19 | 1 | 1 | 1 | 358 | 1204 |
