# Supplementary material for: Push by a net, pull by a cow: can zooprophylaxis enhance the impact of insecticide treated bed nets on malaria control?
Source: Parasit Vectors. 2014 Jan 28;7:52. doi: 10.1186/1756-3305-7-52 (PMC3917899; doi:10.1186/1756-3305-7-52)
Supplement: Additional file 4: Table S3 — Poisson GLMM selection for the abundance of all Anopheles species combined. [file 1756-3305-7-52-S4.docx]

| Table S3. Poisson GLMM selection for the abundance of all *Anopheles* species combined. | | |
| --- | --- | --- |
| Fixed factors | AIC | ΔAIC |
| Cattle 150m, Goats/Sheep 20m, ITNs in use, Residents, Houses 50m, Ephemeral 500m, Permanent, Month, House size | 1117.2 | 7.6 |
| Cattle 150m, Goats/Sheep 20m, ITNs in use, Residents, Houses 50m, Permanent, Month, House size | 1115.3 | 5.7 |
| Cattle 150m, Goats/Sheep 20m, ITNs in use, Residents, Permanent, Month, House size | 1113.5 | 3.9 |
| Cattle 150m, Goats/Sheep 20m, ITNs in use, Residents, Month, House size | 1111.6 | 2.0 |
| Goats/Sheep 20m, ITNs in use, Residents, Month, House size | 1111.2 | 1.6 |
| ***Goats/Sheep 20m, ITNs in use, Residents, House size*** | ***1109.6*** | ***0.0*** |
| Each row presents the fixed factors for each model. Collection date and household were the random effects. | | |
| The model with the lowest AIC is shown in boldface italic type |  |  |
