# Supplementary material for: Push by a net, pull by a cow: can zooprophylaxis enhance the impact of insecticide treated bed nets on malaria control?
Source: Parasit Vectors. 2014 Jan 28;7:52. doi: 10.1186/1756-3305-7-52 (PMC3917899; doi:10.1186/1756-3305-7-52)
Supplement: Additional file 5: Table S4 — Poisson GLMM selection for the abundance of each Anopheles species. [file 1756-3305-7-52-S5.docx]

| Table S4. Poisson GLMM selection for the abundance of each *Anopheles* species. | | |
| --- | --- | --- |
|  |  |  |
| *Anopheles arabiensis* |  |  |
| Fixed factors | AIC | ΔAIC |
| Cattle 150m, Goats/Sheep 20m, ITNs in use, Residents, Houses 50m, Ephemeral 500m, Permanent, Month, House size | 603.7 | 6.8 |
| Cattle 150m, Goats/Sheep 20m, ITNs in use, Houses 50m, Ephemeral 500m, Permanent, Month, House size | 601.8 | 4.8 |
| Cattle 150m, Goats/Sheep 20m, ITNs in use, Houses 50m, Ephemeral 500m, Month, House size | 599.8 | 2.9 |
| Goats/Sheep 20m, ITNs in use, Houses 50m, Ephemeral 500m, Month, House size | 597.9 | 1.0 |
| ***Goats/Sheep 20m, ITNs in use, Houses 50m, Month, House size*** | ***596.9*** | ***0.0*** |
|  |  |  |
| *An.gambiae s.s* |  |  |
| Fixed Factors | AIC | ΔAIC |
| Cattle 150m, Goats/Sheep 150m, ITNs in use, Residents, Houses 50m, Ephemeral 150m, Permanent, Month, House size | 275.8 | 9.6 |
| Cattle 150m, Goats/Sheep 150m, ITNs in use, Residents, Houses 50m, Ephemeral 150m, Month, House size | 273.8 | 7.6 |
| Cattle 150m, ITNs in use, Residents, Houses 50m, Ephemeral 150m, Month, House size | 271.9 | 5.6 |
| ITNs in use, Residents, Houses 50m, Ephemeral 150m, Month, House size | 270.0 | 3.7 |
| ITNs in use, Residents, Houses 50m, Month, House size | 268.1 | 1.8 |
| ***ITNs in use, Residents, Houses 50m, Month*** | ***266.2*** | ***0.0*** |
|  |  |  |
| *An.funesutus s.s.* |  |  |
| Fixed Factors | AIC | ΔAIC |
| Cattle 150m, Goats/Sheep150m, ITNs in use, Residents, Houses 150m, Ephemeral 500m, Permanent, Month, House size | 681.8 | 6.0 |
| Cattle 150m, Goats/Sheep150m, ITNs in use, Residents, Houses 150m, Ephemeral 500m, Permanent, Month | 679.8 | 4.0 |
| Cattle 150m, ITNs in use, Residents, Houses 150m, Ephemeral 500m, Permanent, Month | 678.0 | 2.2 |
| Cattle 150m, ITNs in use, Residents, Houses 150m, Ephemeral 500m, Month | 676.4 | 0.5 |
| ***ITNs in use, Residents, Houses 150m, Ephemeral 500m, Month*** | ***675.8*** | ***0.0*** |
| Each row presents the fixed factors for each model. Collection date and individual household were the random effects. | | |
| The model with the lowest AIC is shown in boldface italic type |  |  |
