# Supplementary material for: Push by a net, pull by a cow: can zooprophylaxis enhance the impact of insecticide treated bed nets on malaria control?
Source: Parasit Vectors. 2014 Jan 28;7:52. doi: 10.1186/1756-3305-7-52 (PMC3917899; doi:10.1186/1756-3305-7-52)
Supplement: Additional file 6: Table S5 — Binomial GLMM selection for Anopheles spp. mosquito feeding on humans over other bloodmeal sources. [file 1756-3305-7-52-S6.docx]

| Table S5. Binomial GLMM selection for *Anopheles* spp. mosquito feeding on humans over other bloodmeal sources. | | |
| --- | --- | --- |
| Fixed Factors | AIC | ΔAIC |
| Cattle 500m, Goats/Sheep 500m, ITNs in use, Residents, Houses 50m, Ephemeral 450m, Permanent, Month, House size | 339.4 | 5.0 |
| Goats/Sheep 500m, ITNs in use, Residents, Houses 50m, Ephemeral 450m, Permanent, Month, House size | 337.5 | 3.2 |
| Goats/Sheep 500m, ITNs in use, Residents, Houses 50m, Ephemeral 450m, Month, House size | 335.5 | 1.2 |
| ***Goats/Sheep 500m, ITNs in use, Residents, Houses 50m, Month, House size*** | ***334.4*** | ***0.0*** |
| Each row presents the fixed factors for each model. Collection date and household were the random effects in all models. | | |
| The model with the lowest AIC is shown in boldface italic type. | | |
