# Supplementary material for: Push by a net, pull by a cow: can zooprophylaxis enhance the impact of insecticide treated bed nets on malaria control?
Source: Parasit Vectors. 2014 Jan 28;7:52. doi: 10.1186/1756-3305-7-52 (PMC3917899; doi:10.1186/1756-3305-7-52)
Supplement: Additional file 7: TableS 6 — Binomial GLMM selection for each Anopheles species feeding on humans over other bloodmeal sources. [file 1756-3305-7-52-S7.docx]

| TableS6. Binomial GLMM selection for each *Anopheles* species feeding on humans over other bloodmeal sources. | | |
| --- | --- | --- |
|  |  |  |
| *Anopheles arabiensis* |  |  |
| Fixed Factors | AIC | ΔAIC |
| Cattle 500m, Goats/Sheep 500m, ITNs in use, Residents, Houses 150m, Ephemeral 500m, Permanent, Month, House size | 151.2 | 5.5 |
| Cattle 500m, Goats/Sheep 500m, Residents, Houses 150m, Ephemeral 500m, Permanent, Month, House size | 149.2 | 3.5 |
| Cattle 500m, Goats/Sheep 500m, Residents, Houses 150m, Ephemeral 500m, Permanent, Month | 147.2 | 1.5 |
| Cattle 500m, Goats/Sheep 500m, Residents, Houses 150m, Ephemeral 500m, Month | 145.8 | 0.1 |
| ***Cattle 500m, Goats/Sheep 500m, Residents, Houses 150m, Month*** | ***145.7*** | ***0.0*** |
| Each row presents the fixed factors for each model and collection date was the random effect. | | |
|  |  |  |
| *An.gambiae s.s.* | | |
| Fixed Factors | AIC | ΔAIC |
| Cattle 20m, Goats/Sheep 20m, ITNs in use, Residents, Houses 50m, Ephemeral 450m, Permanent, Month, House size | 104.8 | 10.9 |
| Cattle 20m, Goats/Sheep 20m, ITNs in use, Residents, Houses 50m, Ephemeral 450m, Month, House size | 102.8 | 8.9 |
| Cattle 20m, Goats/Sheep 20m, ITNs in use, Residents, Ephemeral 450m, Month, House size | 100.8 | 7.0 |
| Cattle 20m, Goats/Sheep 20m, ITNs in use, Residents, Ephemeral 450m, Month | 98.9 | 5.1 |
| Goats/Sheep 20m, ITNs in use, Residents, Ephemeral 450m, Month | 97.0 | 3.2 |
| Goats/Sheep 20m, ITNs in use, Residents, Ephemeral 450m | 95.4 | 1.6 |
| ***Goats/Sheep 20m, ITNs in use, Residents*** | ***93.8*** | ***0.0*** |
| Each row presents the fixed factors for each model and collection date was a random effect. | | |
|  |  |  |
| *An.funestus s.s.* | | |
| Fixed Factors | AIC | ΔAIC |
| Cattle 50m, Goats/Sheep 300m, ITNs in use, Residents, Houses 50m, Ephemeral 200m, Permanent, Month, House size | 142.8 | 3.8 |
| Cattle 50m, Goats/Sheep 300m, ITNs in use, Residents, Houses 50m, Ephemeral 200m, Month, House size | 140.9 | 1.8 |
| Cattle 50m, Goats/Sheep 300m, ITNs in use, Residents, Ephemeral 200m, Month, House size | 140.4 | 1.4 |
| ***Cattle 50m, Goats/Sheep 300m, ITNs in use, Residents, Ephemeral 200m, Month*** | ***139.0*** | ***0.0*** |
| Each row presents the fixed factors for each model and household was the random effect. | | |
|  |  |  |
