# Supplementary material for: Push by a net, pull by a cow: can zooprophylaxis enhance the impact of insecticide treated bed nets on malaria control?
Source: Parasit Vectors. 2014 Jan 28;7:52. doi: 10.1186/1756-3305-7-52 (PMC3917899; doi:10.1186/1756-3305-7-52)
Supplement: Additional file 8: Table S7 — Poisson rate GLMM selection for the sporozoite rate of all Anopheles spp. combined. [file 1756-3305-7-52-S8.docx]

| Table S7. Poisson rate GLMM selection for the sporozoite rate of all *Anopheles* spp. combined. | | |
| --- | --- | --- |
| Fixed Factors | AIC | ΔAIC |
| Cattle blood index, Goats/Sheep blood index, ITN coverage, Ephemeral 250m, Permanent, Month, House size | 80.1 | 4.9 |
| Cattle blood index, Goats/Sheep blood index, ITN coverage, Ephemeral 250m, Permanent, Month | 78.4 | 3.1 |
| Cattle blood index, Goats/Sheep blood index, ITN coverage, Ephemeral 250m, Month | 76.7 | 1.5 |
| Cattle blood index, Goats/Sheep blood index, Ephemeral 250m, Month | 76.7 | 1.4 |
| Cattle blood index, Goats/Sheep blood index, Ephemeral 250m | 76.1 | 0.9 |
| ***Cattle blood index, Ephemeral 250m*** | ***75.2*** | ***0.0*** |
| Each row presents the fixed factors for each model. Collection date and household were random effects in all models. The model with the lowest AIC is shown in boldface italic type. For the sporozoite rate the numerator where the sporozoite positive mosquitoes and the denominator all gravid and blood fed mosquitoes. | | |
