# Supplementary material for: Push by a net, pull by a cow: can zooprophylaxis enhance the impact of insecticide treated bed nets on malaria control?
Source: Parasit Vectors. 2014 Jan 28;7:52. doi: 10.1186/1756-3305-7-52 (PMC3917899; doi:10.1186/1756-3305-7-52)
Supplement: Additional file 9: Table S8 — Poisson rate GLMM selection for the sporozoite rate of Anopheles funestus s.s.. [file 1756-3305-7-52-S9.docx]

| Table S8. Poisson rate GLMM selection for the sporozoite rate of *Anopheles funestus s.s..* | | |
| --- | --- | --- |
| Fixed Factors | AIC | ΔAIC |
| Non-human index, ITN coverage, Ephemeral 250m, Permanent, Month, House size | 58.5 | 8.4 |
| Non-human index, ITN coverage, Ephemeral 250m, Permanent, Month | 56.5 | 6.5 |
| Non-human index, Ephemeral 250m, Permanent, Month | 54.8 | 4.7 |
| Non-human index, Ephemeral 250m, Permanent | 52.9 | 2.8 |
| Non-human index, Ephemeral 250m | 51.3 | 1.2 |
| ***Ephemeral 250m*** | ***50.1*** | ***0.0*** |
| Each row presents the fixed factors for each model. Collection date was the only random effects in all models. The model with the lowest AIC is shown in boldface italic type. For the sporozoite rate the numerator where the sporozoite positive *An.funestus s.s.* mosquitoes and the denominator all gravid and bloodfed *An.funestus s.s.* mosquitoes. | | |
